# ATP–P2X3 Signaling as a Shared Neural Sensitization Pathway in Endometriosis and Irritable Bowel Syndrome: Mechanisms and Therapeutic Implications

**DOI:** 10.3390/biomedicines14091891

**Published:** 2026-08-25

**Authors:** My Anh Duong, Prakash V. A. K. Ramdass

**Affiliations:** Department of Public Health and Preventive Medicine, School of Medicine, St. George’s University, St. George P.O. Box 7, Grenada; mduong1@sgu.edu

**Keywords:** ATP, P2X3 receptor, purinergic signaling, endometriosis, irritable bowel syndrome, visceral hypersensitivity, chronic pelvic pain, neuroimmune crosstalk, P2X3 antagonists

## Abstract

Endometriosis and irritable bowel syndrome (IBS) are chronic pain disorders that frequently coexist and share key pathophysiological features, including neuroinflammation, visceral hypersensitivity, peripheral sensitization, and central sensitization. Emerging evidence suggests that extracellular adenosine triphosphate (ATP)-mediated activation of the P2X3 receptor is a common mechanism driving persistent nociceptive signaling in both conditions. This narrative review examines the role of ATP–P2X3 signaling in the pathogenesis of endometriosis and IBS, highlighting its involvement in neuroimmune crosstalk, dorsal root ganglion plasticity, and cross-organ sensitization. We summarize experimental and clinical evidence supporting P2X3 as a therapeutic target and discuss the development of selective P2X3 antagonists, including gefapixant, eliapixant, sivopixant, and camlipixant. Although these agents have shown clinical benefit in refractory chronic cough, their application to endometriosis and IBS remains largely unexplored. Current evidence is predominantly preclinical, underscoring the need for biomarker-driven translational studies and clinical trials. Overall, ATP–P2X3 signaling represents a promising shared mechanistic pathway linking endometriosis and IBS and a potential target for the development of precision, nonopioid therapies for chronic pelvic and visceral pain.

## 1. Introduction

Endometriosis and irritable bowel syndrome (IBS) are common chronic disorders that substantially impair quality of life, contribute to significant healthcare utilization, and impose considerable socioeconomic burden worldwide [1]. Endometriosis affects approximately 10% of women of reproductive age and is characterized by the ectopic implantation of endometrial-like tissue, resulting in chronic pelvic pain, dysmenorrhea, dyspareunia, infertility, and gastrointestinal symptoms [2]. Similarly, IBS is one of the most prevalent disorders of gut–brain interaction, affecting an estimated 5–10% of the global population and presenting with recurrent abdominal pain, altered bowel habits, bloating, and visceral hypersensitivity [3,4]. Although they are distinct clinical entities, accumulating evidence suggests that these conditions frequently coexist and may share overlapping mechanisms of chronic pain generation [5].

The coexistence of endometriosis and IBS is increasingly recognized in clinical practice [6]. Women with endometriosis are significantly more likely to meet diagnostic criteria for IBS than the general population, and gastrointestinal symptoms frequently persist even after surgical treatment of endometriotic lesions [7]. These observations suggest the presence of shared biological mechanisms, although the pathways responsible for symptom persistence remain incompletely understood [8].

Chronic pain in both endometriosis and IBS is believed to arise from complex interactions among peripheral inflammation, immune activation, visceral hypersensitivity, and central nervous system sensitization [9,10]. Multiple molecular mediators, including transient receptor potential vanilloid 1 (TRPV1), nerve growth factor (NGF), calcitonin gene-related peptide (CGRP), substance P, acid-sensing ion channels (ASICs), and purinergic receptors, have been implicated in nociceptive signaling [11,12,13,14,15]. Among these pathways, purinergic signaling through the P2X3 receptor has emerged as a particularly compelling candidate because of its well-established role in sensory neuron activation, inflammatory pain transmission, and peripheral sensitization [16]. Extracellular adenosine triphosphate (ATP), released during tissue injury, inflammation, hypoxia, or cellular stress, functions as an important extracellular signaling molecule by activating P2X receptors expressed on primary sensory neurons, immune cells, and peripheral nerve terminals, thereby promoting neuronal excitability and amplifying nociceptive transmission [17,18]. Among the seven P2X receptor subtypes, P2X3 plays a central role in the initiation and maintenance of inflammatory and neuropathic pain [19]. Experimental studies have demonstrated that increased ATP release and P2X3 receptor activation contribute to visceral hypersensitivity in gastrointestinal disorders and chronic pelvic pain models, highlighting the receptor as a potential therapeutic target [20].

The development of selective P2X3 antagonists has renewed interest in purinergic signaling as a clinically actionable pathway [21,22,23,24]. Although these agents have primarily been investigated for refractory chronic cough, their ability to attenuate sensory neuron hyperexcitability suggests potential applications in chronic visceral pain disorders, including endometriosis and IBS [25].

Nevertheless, the current evidence remains heterogeneous and is derived predominantly from experimental and animal studies. Thus, this narrative review critically examines the available experimental and clinical evidence supporting ATP–P2X3 signaling as a shared mechanism linking endometriosis and IBS. We evaluated the therapeutic potential of P2X3 antagonists, discussed the limitations of the current evidence, and highlighted priorities for future translational and clinical research.

## 2. Clinical Overlap Between Endometriosis and IBS

**Figure 1:**
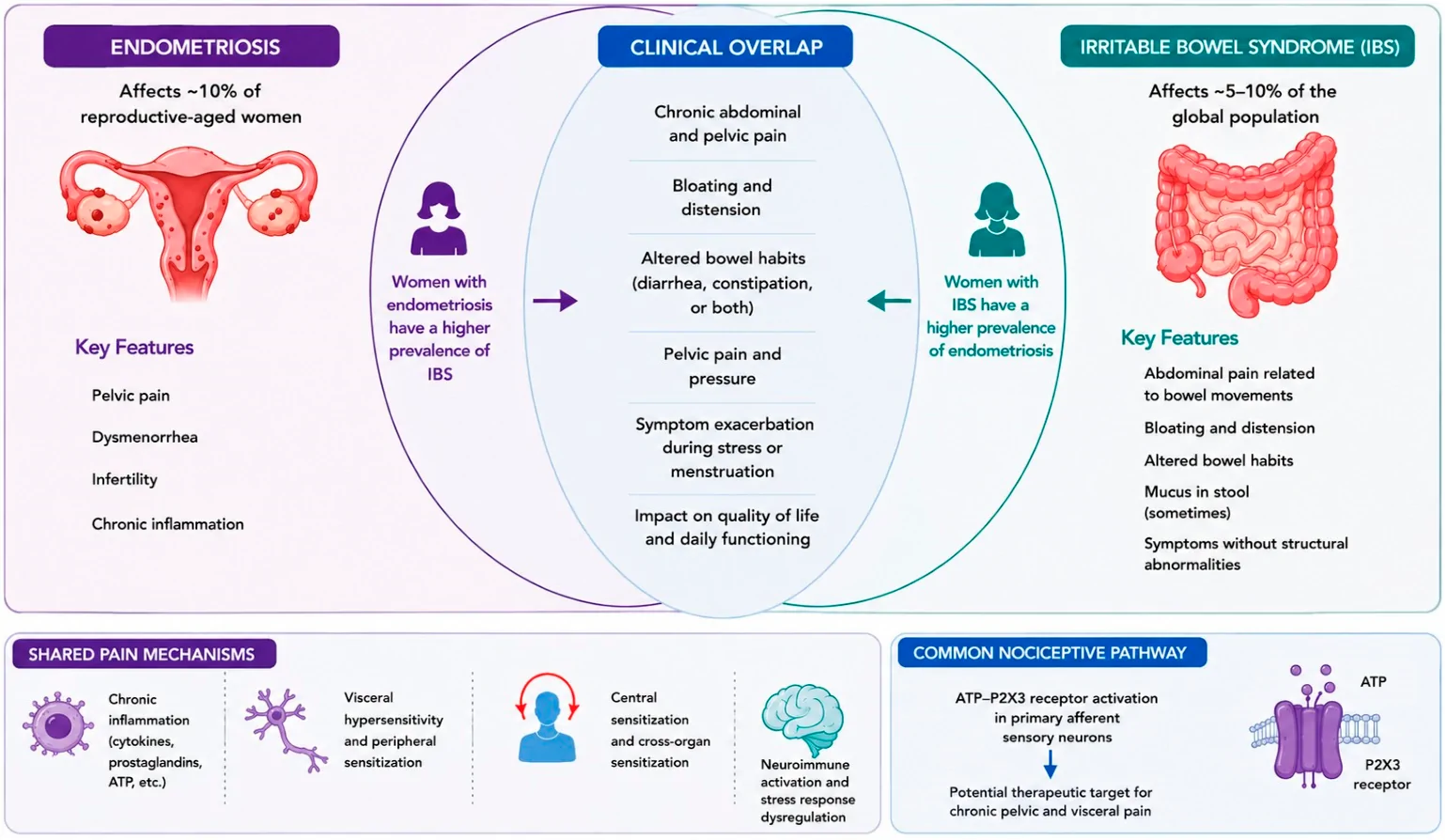
Clinical overlap between endometriosis and IBS. Endometriosis and IBS share multiple clinical manifestations, including chronic pelvic and abdominal pain, bloating, altered bowel habits, and symptom exacerbation during stress or menstruation. Their frequent coexistence is associated with visceral hypersensitivity, peripheral and central sensitization, and neuroimmune dysregulation, suggesting convergence on common nociceptive pathways. ATP-mediated activation of P2X3 receptors has emerged as a potential mechanistic link underlying chronic pelvic and visceral pain, supporting the rationale for targeting P2X3 signaling as a novel therapeutic strategy for both disorders.

Endometriosis and IBS frequently coexist and exhibit substantial clinical overlap (see Figure 1), often making accurate diagnosis challenging [7]. Both disorders commonly present with chronic pelvic and abdominal pain, bloating, altered bowel habits, dyschezia, and symptom exacerbation during periods of physiological stress or menstruation, leading to frequent misdiagnosis and delays in appropriate management [8,26]. Women with endometriosis have a significantly higher prevalence of IBS than the general population, highlighting a close clinical association between these conditions [7]. Beyond their overlapping symptomatology, both disorders are characterized by visceral hypersensitivity [27,28], peripheral and central sensitization [29,30], and neuroimmune dysregulation [31,32], suggesting shared mechanisms of chronic pain generation. This convergence has drawn increasing attention toward common nociceptive pathways, particularly ATP-mediated activation of P2X3 receptors, which may contribute to persistent sensory neuron activation and represent a promising therapeutic target for treating chronic pelvic and visceral pain in patients with endometriosis and IBS.

## 3. ATP–P2X3 Purinergic Signaling in Chronic Pain

### 3.1. Purinergic Signaling and ATP Release

Purinergic signaling is a fundamental mechanism of intercellular communication that regulates inflammation, nociception, and tissue homeostasis [33]. Under physiological conditions, intracellular ATP functions primarily as the cell’s energy currency. However, tissue injury, inflammation, hypoxia, mechanical stress, and cellular damage trigger the extracellular release of ATP through pannexin and connexin hemichannels or from damaged cells [34]. Once released, ATP acts as a danger-associated molecular pattern (DAMP), activating purinergic receptors expressed on sensory neurons, immune cells, epithelial cells, and glial cells [35].

Extracellular ATP contributes to neurogenic inflammation by stimulating the release of pro-inflammatory cytokines, chemokines, and neuropeptides while simultaneously increasing the excitability of primary afferent neurons [34]. Although ATP activates both metabotropic P2Y receptors and ionotropic P2X receptors, increasing evidence indicates that P2X3 receptors are particularly important mediators of chronic inflammatory and visceral pain [36]. This makes them attractive therapeutic targets for disorders characterized by persistent nociceptive signaling [37].

Purinergic dysregulation encompasses a broad spectrum of abnormalities in extracellular nucleotide metabolism and receptor signaling rather than activation of a single receptor [38]. Reduced ectonucleotidase activity, increased extracellular ATP accumulation, and altered expression of multiple purinergic receptors collectively contribute to a pro-inflammatory and pronociceptive microenvironment. Within this broader network, activation of P2X3 receptors on primary sensory neurons represents a downstream mechanism that specifically mediates peripheral nociceptor activation and pain transmission [39]. Thus, while purinergic dysregulation contributes to disease pathophysiology, P2X3-mediated nociception represents one of its principal functional consequences.

### 3.2. Structure and Physiological Function of the P2X3 Receptor

The P2X receptor family comprises seven ATP-gated ion channel subtypes (P2X1–P2X7), with P2X3 being one of the principal receptors involved in nociception [40]. P2X3 receptors are predominantly expressed on small- and medium-diameter sensory neurons within the dorsal root, trigeminal, and nodose ganglia, where they detect extracellular ATP released during tissue injury and inflammation [13]. Functional receptors exist as homotrimeric P2X3 channels or heterotrimeric P2X2/3 receptors, both of which are highly permeable to sodium and calcium ions [40].

Activation of P2X3 receptors produces rapid membrane depolarization, calcium influx, and action potential generation, facilitating transmission of nociceptive signals from peripheral tissues to the central nervous system [25]. Beyond neuronal excitation, P2X3 activation promotes the release of neurotransmitters and neuropeptides that further amplify inflammatory signaling [41]. Persistent receptor activation therefore contributes to sustained neuronal hyperexcitability, a hallmark of chronic pain disorders [36]. The structural distinction between homomeric P2X3 and heteromeric P2X2/3 receptors has guided the development of selective antagonists, with newer agents designed to minimize P2X2/3 inhibition to reduce taste-related adverse effects [42].

### 3.3. P2X3 Receptors in Peripheral and Central Sensitization

Peripheral sensitization, characterized by increased nociceptor responsiveness following tissue injury or inflammation, and central sensitization, resulting from enhanced excitability within spinal pain pathways, are both amplified by ATP–P2X3 signaling [29]. Inflammatory mediators such as prostaglandins, NGF, bradykinin, histamine, and pro-inflammatory cytokines promote ATP release and P2X3 receptor upregulation, creating a positive feedback loop that enhances synaptic transmission and promotes chronic visceral hypersensitivity [43]. Experimental models of endometriosis and IBS consistently demonstrate increased ATP release, elevated P2X3 receptor expression, and enhanced sensory neuron excitability, supporting dysregulated ATP–P2X3 signaling as a shared mechanism of chronic pelvic and visceral pain and highlighting selective P2X3 inhibition as a promising therapeutic strategy [44].

## 4. Role of ATP–P2X3 Signaling in Endometriosis

### 4.1. Neuroinflammation and Peripheral Nerve Remodeling

Endometriosis is increasingly recognized as a neuroinflammatory disorder characterized by chronic activation of immune and sensory pathways [45]. In endometriosis, purinergic dysregulation extends beyond P2X3 receptor activation and includes impaired extracellular ATP metabolism, altered ectonucleotidase activity, and activation of multiple purinergic receptor subtypes that collectively sustain inflammation and tissue remodeling [46]. Ectopic endometrial lesions recruit macrophages, mast cells, and other immune cells that release pro-inflammatory cytokines, prostaglandins, nerve growth factor (NGF), and chemokines, creating a persistent inflammatory microenvironment that promotes neuroangiogenesis, peripheral nerve remodeling, and increased nociceptor excitability [47]. Sustained inflammation lowers nociceptor activation thresholds, leading to peripheral sensitization, while persistent nociceptive input induces DRG plasticity and central sensitization, mechanisms that contribute to chronic pelvic pain and symptom persistence even after surgical excision of endometriotic lesions [48]. Importantly, pain severity does not consistently correlate with lesion burden alone, and emerging evidence suggests that lesion location, the extent of adhesions, and central pain sensitization may be more closely associated with symptom severity [49,50].

### 4.2. ATP Release and P2X3 Receptor Expression

**Figure 2:**
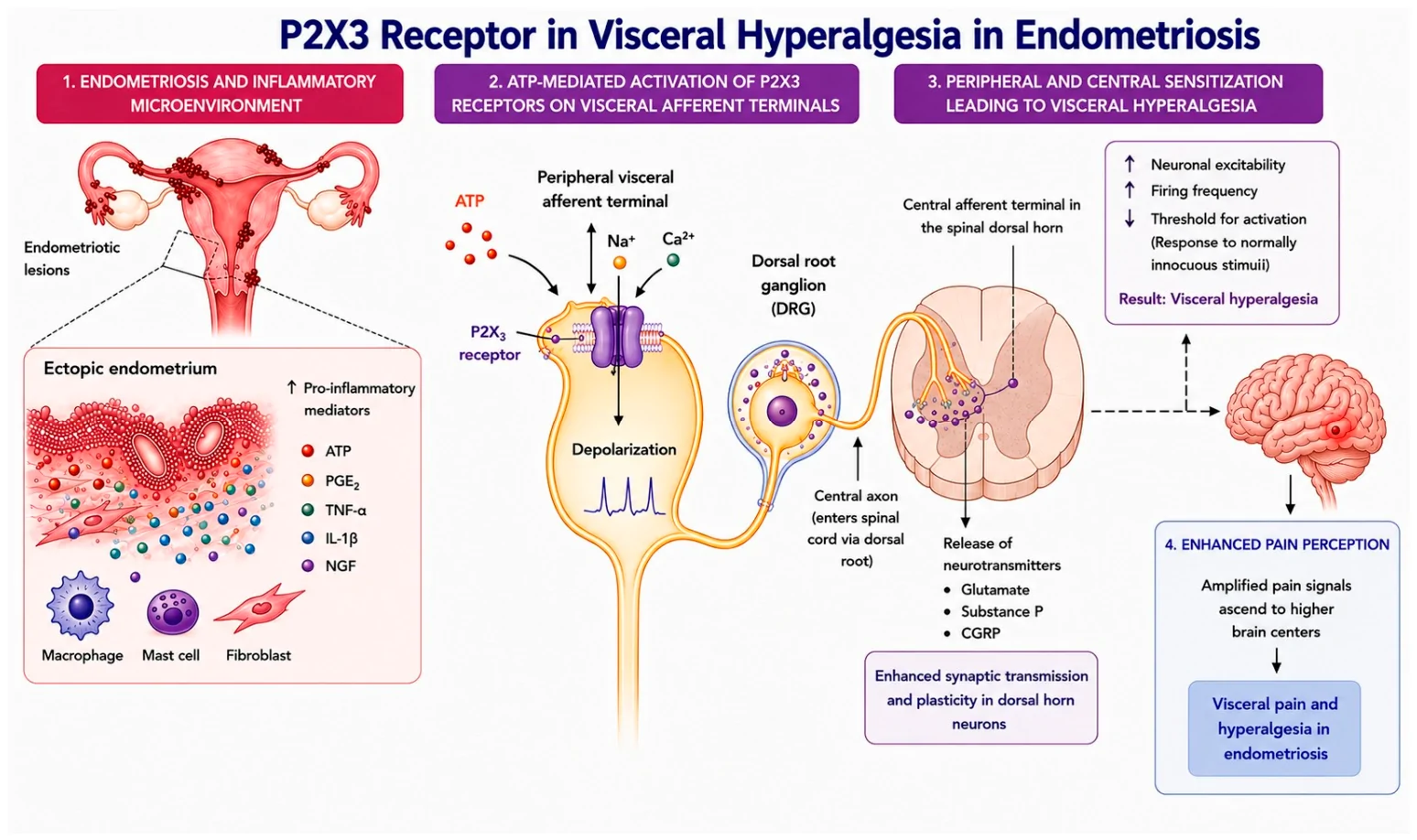
P2X3 receptor-mediated peripheral and central sensitization in endometriosis. (**1**) Ectopic endometrial lesions create an inflammatory microenvironment characterized by ATP release and increased production of sensitizing mediators, including prostaglandin E_2_ (PGE_2_), tumor necrosis factor-α (TNF-α), interleukin-1β (IL-1β), and nerve growth factor (NGF), involving macrophages, mast cells, and fibroblasts. (**2**) ATP activates P2X3 receptors on peripheral visceral afferent terminals, promoting Na^+^ and Ca^2+^ influx, membrane depolarization, and action-potential generation. These nociceptive signals are transmitted along primary afferent neurons whose cell bodies reside in the dorsal root ganglia (DRG). (**3**) Persistent afferent input increases the release of glutamate, substance P, and calcitonin gene-related peptide (CGRP) from central afferent terminals in the spinal dorsal horn, enhancing synaptic transmission and neuronal plasticity. The resulting increases in neuronal excitability and firing frequency, together with a reduced activation threshold, contribute to peripheral and central sensitization and visceral hyperalgesia. (**4**) Amplified nociceptive signals ascend to higher brain centers, resulting in enhanced visceral pain perception in endometriosis. P2X3 receptor antagonism may attenuate peripheral afferent activation and downstream sensitization, representing a potential therapeutic approach.

Inflammation and tissue injury within endometriotic lesions promote the extracellular release of ATP from damaged epithelial, stromal, immune, and sensory nerve cells, where it functions as a danger-associated molecular pattern that activates ATP-gated P2X receptors on primary afferent neurons, amplifying inflammatory pain signaling [51]. Among these receptors, P2X3 is of particular interest because of its predominant expression on nociceptive sensory neurons and its established role in chronic inflammatory pain [52]. Experimental studies demonstrate increased P2X3 receptor expression in DRG innervating endometriotic lesions, leading to enhanced neuronal excitability, calcium influx, neurotransmitter release, visceral hypersensitivity, and sustained nociceptive transmission, thereby establishing a positive feedback loop that perpetuates chronic pelvic pain [53]. The proposed ATP–P2X3 signaling pathway underlying peripheral and central sensitization in endometriosis is summarized in Figure 2.

## 5. Role of ATP–P2X3 Signaling in IBS

### 5.1. Visceral Hypersensitivity

Visceral hypersensitivity is a defining feature of IBS and is considered a major contributor to chronic abdominal pain [54]. Patients with IBS exhibit heightened perception of physiological intestinal stimuli, reflecting increased excitability of primary afferent neurons and altered pain processing within the gut–brain axis [55]. Persistent low-grade inflammation, epithelial barrier dysfunction, and repeated luminal stimulation promote the release of ATP and other inflammatory mediators that sensitize nociceptive pathways [34]. Activation of P2X3 receptors on visceral afferent neurons lowers activation thresholds, facilitating exaggerated pain responses and contributing to the maintenance of chronic visceral hypersensitivity [56].

### 5.2. Enteric Neuroimmune Interactions

**Figure 3:**
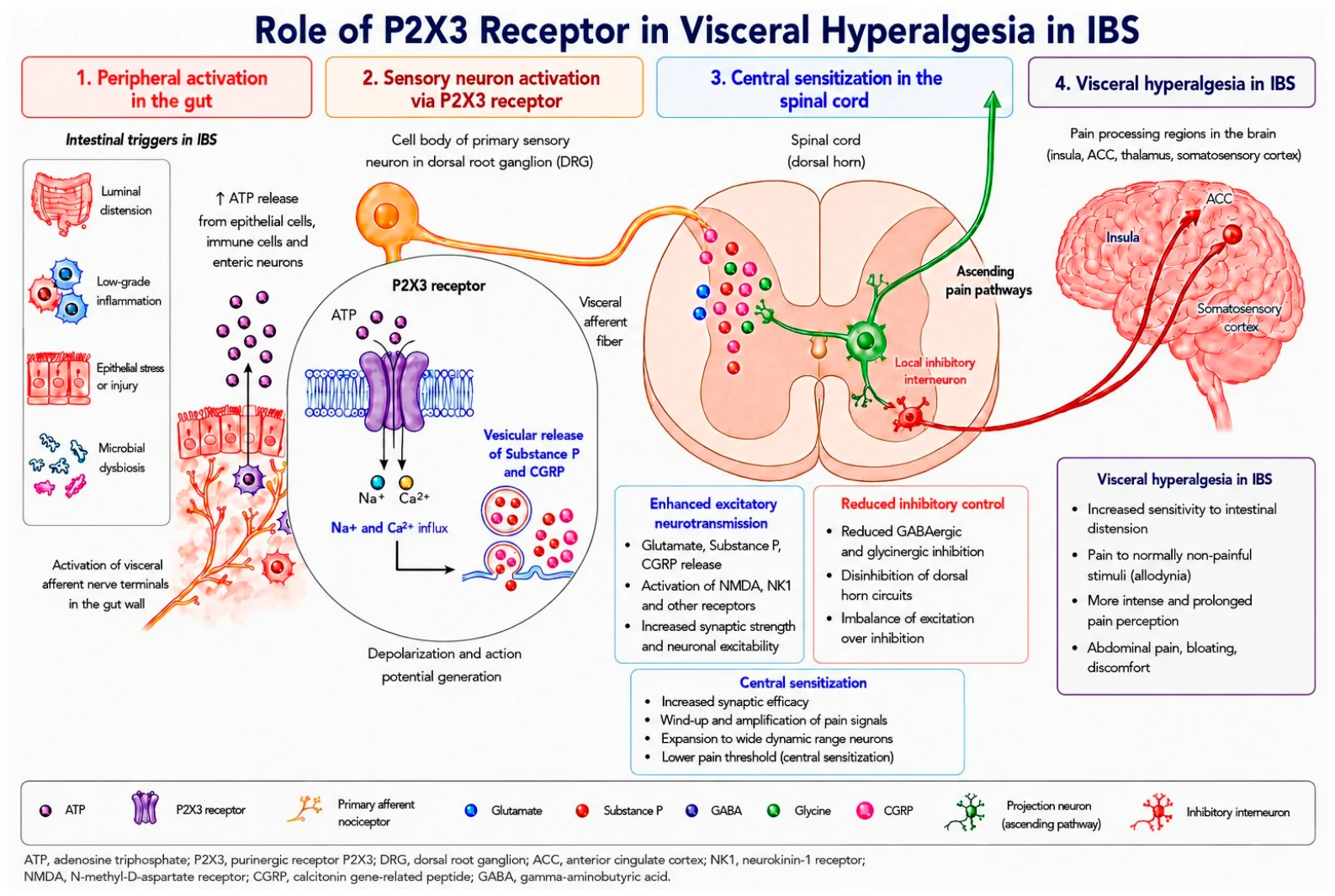
Schematic representation of P2X3 purinergic receptor-mediated neural sensitization in IBS. (**1**) Peripheral stressors in IBS, including luminal distension, low-grade inflammation, epithelial injury, and microbial dysbiosis, promote extracellular ATP release from epithelial, immune, and enteroendocrine cells within the gut microenvironment. (**2**) ATP released in the gut activates P2X3 receptors expressed on the peripheral terminals of visceral primary afferent nociceptive neurons. Activation of these receptors depolarizes the nerve terminals, generating action potentials that are transmitted to the dorsal horn of the spinal cord. Increased release of excitatory neurotransmitters, including glutamate, substance P, and CGRP, in the dorsal horn of the spinal cord amplifies nociceptive signaling. This enhanced excitatory transmission contributes to central sensitization and ultimately to visceral hyperalgesia in patients with IBS. (**3**) Sustained nociceptive signaling enhances excitatory neurotransmitter release, including glutamate, substance P, and calcitonin gene-related peptide (CGRP), within the spinal dorsal horn, contributing to central sensitization. (**4**) Amplified pain transmission to higher brain regions, such as the insula, anterior cingulate cortex (ACC), and thalamus, results in visceral hyperalgesia characterized by abdominal discomfort, bloating, and altered pain perception in IBS. Therapeutically, antagonism or desensitization of P2X3 receptors may attenuate nociceptive signaling and reduce visceral pain perception.

IBS is increasingly recognized as a neuroimmune disorder in which bidirectional communication between immune cells, enteric neurons, and the intestinal epithelium sustains chronic pain [57]. Increased mast cell activation, macrophage infiltration, and elevated levels of inflammatory mediators, including histamine, serotonin, cytokines, prostaglandins, and ATP, have been reported in subsets of patients with IBS [58]. Extracellular ATP released during intestinal inflammation activates P2X3 receptors expressed on sensory neurons, amplifying nociceptive signaling and promoting neurogenic inflammation. Interactions between ATP–P2X3 signaling and other pain mediators, including NGF, TRPV1, and CGRP, further enhance neuronal excitability and contribute to persistent abdominal pain [59]. The proposed ATP–P2X3 signaling pathway underlying visceral hypersensitivity in IBS is summarized in Figure 3.

## 6. Shared Neuroimmune Mechanisms Linking Endometriosis and IBS

### 6.1. Cross-Organ Sensitization

The frequent coexistence of endometriosis and IBS suggests that chronic pain may arise through shared neurobiological mechanisms rather than independent disease processes [7]. One proposed mechanism is cross-organ sensitization, whereby persistent nociceptive input from one pelvic organ enhances pain perception in adjacent organs through convergent neural pathways. In endometriosis, continuous stimulation from ectopic lesions may sensitize visceral afferents supplying the gastrointestinal tract, contributing to IBS-like symptoms [60]. Conversely, chronic intestinal inflammation and visceral hypersensitivity may amplify pelvic pain through similar mechanisms. ATP released during tissue injury and inflammation is likely to contribute to this process by activating P2X3 receptors on shared sensory neurons, thereby facilitating persistent nociceptive transmission between pelvic organs [61].

### 6.2. DRG Plasticity

The DRG serves as a critical integration center for sensory input from pelvic and abdominal organs. Sustained activation of primary afferent neurons induces structural and functional plasticity within the DRG, including increased expression of ion channels, neurotransmitters, and purinergic receptors [62]. Experimental models have demonstrated upregulation of P2X3 receptors in DRG neurons following chronic inflammatory stimulation, resulting in enhanced neuronal excitability and prolonged pain signaling [43]. These changes may underlie the transition from acute inflammation to chronic pelvic and visceral pain and provide a mechanistic basis for the persistent symptoms observed in both endometriosis and IBS.

### 6.3. Neuroimmune Crosstalk

Neuroimmune interactions play a central role in maintaining chronic pain in both disorders. Activated macrophages, mast cells, T lymphocytes, and other immune cells release cytokines, chemokines, prostaglandins, and ATP, which sensitize peripheral nociceptors and perpetuate inflammation [58]. In turn, activated sensory neurons locally release neuropeptides, including substance P and CGRP, further promoting immune cell activation and neurogenic inflammation [63]. This bidirectional communication establishes a self-sustaining inflammatory cycle that enhances neuronal excitability and chronic pain [64]. ATP-mediated activation of P2X3 receptors represents an important interface between immune activation and nociceptive signaling, positioning purinergic signaling as a potential therapeutic target capable of interrupting this pathogenic feedback loop [64].

### 6.4. Interactions with Other Pain Pathways (TRPV1, NGF, CGRP, ASICs)

**Figure 4:**
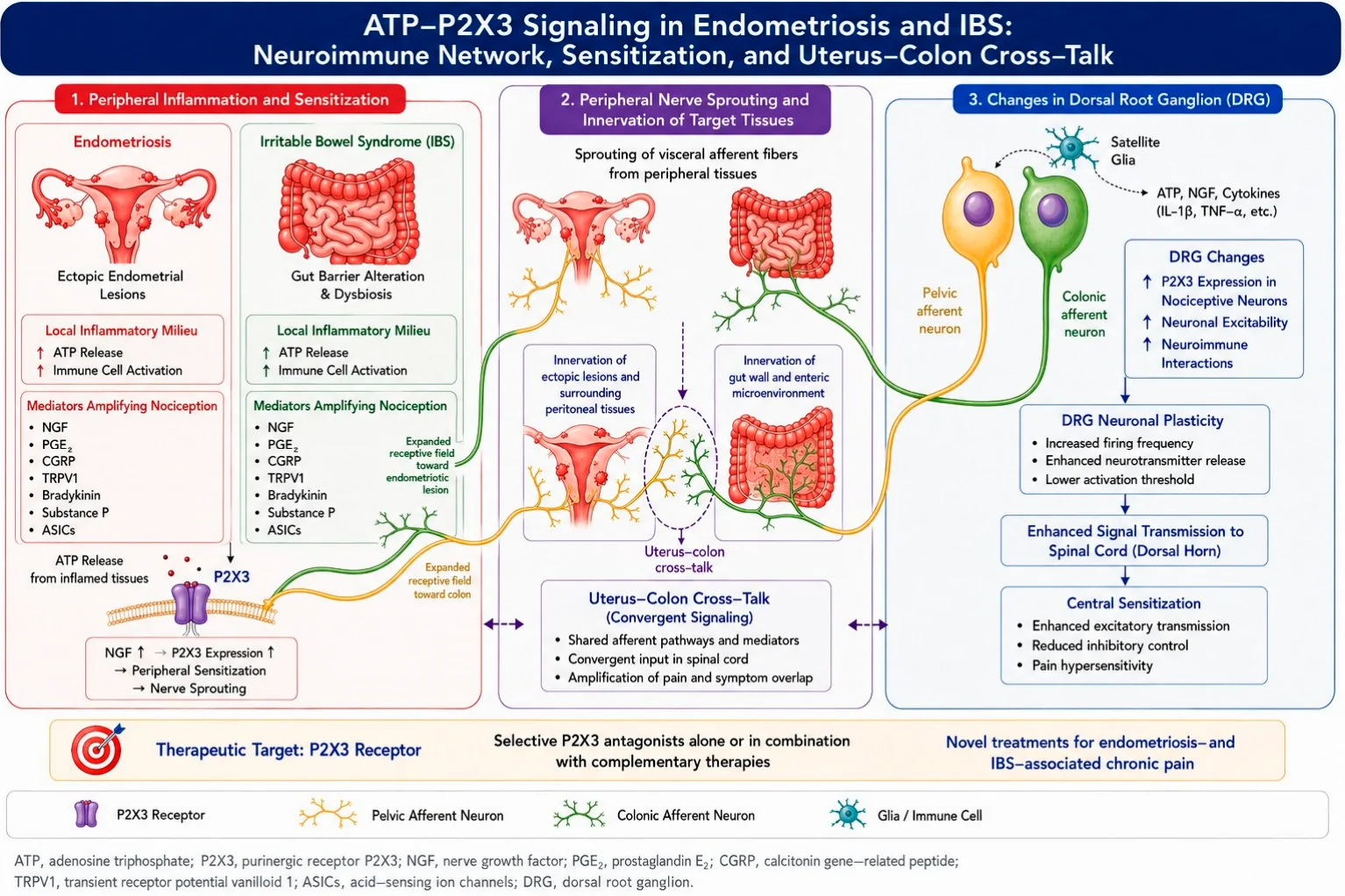
ATP–P2X3 signaling in endometriosis and irritable bowel syndrome: neuroimmune sensitization and uterus–colon cross-talk. (**1**) In endometriosis, ectopic endometrial lesions generate a local inflammatory milieu characterized by increased ATP release and immune-cell activation. In irritable bowel syndrome (IBS), gut-barrier alterations and dysbiosis similarly promote mucosal inflammation, ATP release, and immune activation. Nerve growth factor (NGF), prostaglandin E_2_ (PGE_2_), calcitonin gene-related peptide (CGRP), transient receptor potential vanilloid 1 (TRPV1), bradykinin, substance P, and acid-sensing ion channels (ASICs) amplify nociceptive signaling. ATP activates P2X3 receptors on visceral afferent terminals, while increased NGF signaling may enhance P2X3 expression, peripheral sensitization, and nerve sprouting. (**2**) Sprouting visceral afferent fibers innervate ectopic lesions and surrounding peritoneal tissues in endometriosis and the gut wall and enteric microenvironment in IBS. Shared afferent pathways and convergent spinal inputs facilitate bidirectional uterus–colon cross-talk, amplifying pain and contributing to overlapping pelvic and gastrointestinal symptoms. (**3**) Sustained nociceptive input induces dorsal root ganglion (DRG) plasticity, including increased P2X3 expression, neuronal excitability, firing frequency, and neurotransmitter release, together with a reduced activation threshold. Interactions among nociceptive neurons, satellite glial cells, and inflammatory mediators—including ATP, NGF, interleukin-1β, and tumor necrosis factor-α—further reinforce sensitization. Enhanced transmission to the spinal dorsal horn promotes central sensitization through increased excitatory signaling, reduced inhibitory control, and pain hypersensitivity. Collectively, this pathway identifies P2X3 receptors as a potential therapeutic target for endometriosis- and IBS-associated chronic pain, including through selective P2X3 antagonists alone or in combination with complementary therapies. ATP, adenosine triphosphate; ASICs, acid-sensing ion channels; CGRP, calcitonin gene-related peptide; DRG, dorsal root ganglion; IBS, irritable bowel syndrome; NGF, nerve growth factor; P2X3, purinergic receptor P2X3; PGE_2_, prostaglandin E_2_; TRPV1, transient receptor potential vanilloid 1.

Although ATP–P2X3 signaling plays an important role in chronic pelvic and visceral pain, it functions within a broader neuroimmune network involving mediators such as NGF, TRPV1, CGRP, ASICs, prostaglandins, bradykinin, and substance P, which collectively amplify neuronal activation, peripheral sensitization, and neurogenic inflammation [48,65,66]. In particular, NGF promotes P2X3 receptor expression and sensory nerve sprouting, while TRPV1 and ASICs enhance nociceptor responsiveness to inflammatory stimuli [65,67]. Despite these complex interactions, the selective expression of P2X3 receptors on nociceptive sensory neurons and encouraging preclinical findings make ATP–P2X3 signaling an attractive therapeutic target, warranting further investigation of selective P2X3 antagonists alone or in combination with complementary therapies for endometriosis- and IBS-associated chronic pain [61,68]. The proposed shared ATP–P2X3-mediated pathway underlying cross-organ sensitization, DRG plasticity, neuroimmune crosstalk, and chronic visceral pain is summarized in Figure 4. The key experimental studies supporting ATP–P2X3 signaling as a shared neural sensitization pathway linking endometriosis and IBS are summarized in Table 1.

## 7. Therapeutic Targeting of ATP–P2X3 Signaling

### 7.1. Rationale for P2X3 Inhibition

The selective expression of P2X3 receptors on nociceptive sensory neurons makes them an attractive therapeutic target for chronic pain. Unlike conventional analgesics, P2X3 antagonists selectively inhibit ATP-mediated activation of primary afferent neurons, reducing peripheral sensitization while minimizing the gastrointestinal, cardiovascular, and dependency-related adverse effects associated with nonsteroidal anti-inflammatory drugs (NSAIDs) and opioids [78]. Experimental studies in endometriosis and IBS demonstrate that ATP-mediated P2X3 activation drives persistent nociceptive signaling, DRG hyperexcitability, and visceral hypersensitivity, providing a strong biological rationale for P2X3 inhibition as a novel therapeutic strategy [79]. However, because central sensitization may become established in chronic disease, early intervention or combination therapy may be required to achieve optimal clinical outcomes [21]. Although preclinical studies provided an initial rationale for targeting P2X3 signaling, the phase 2b SCHUMANN trial has since evaluated the selective P2X3 antagonist eliapixant in women with endometriosis-associated pelvic pain [77]. Although the study did not demonstrate significant efficacy over placebo and was terminated early because of safety concerns, it represents the first clinical evaluation of P2X3 antagonism in endometriosis and provides important translational evidence for this therapeutic strategy [77].

### 7.2. Preclinical Studies of P2X3 Antagonists

Numerous experimental studies demonstrate the analgesic potential of selective P2X3 antagonists, with pharmacological inhibition or genetic downregulation of P2X3 reducing nociceptor excitability, ATP-induced neuronal activation, DRG hyperexcitability, and central sensitization [79]. In animal models of endometriosis and IBS, P2X3 blockade attenuates pelvic pain, inflammatory responses, and visceral hypersensitivity, providing consistent preclinical evidence that ATP–P2X3 signaling is a key mediator of chronic pain and a promising therapeutic target [61].

### 7.3. Clinical Development of P2X3 Antagonists

#### 7.3.1. Gefapixant

Gefapixant is the first selective P2X3 receptor antagonist to receive regulatory approval for the treatment of refractory or unexplained chronic cough, validating P2X3 receptors as clinically relevant therapeutic targets [80]. It has received regulatory approval in Japan for refractory chronic cough, although the US FDA issued a Complete Response Letter, highlighting the importance of balancing efficacy and tolerability in P2X3 antagonist development [80]. Clinical trials have demonstrated significant reductions in cough frequency and improvements in patient-reported quality of life [21]. However, because gefapixant also inhibits heteromeric P2X2/3 receptors involved in taste perception, dysgeusia and ageusia have emerged as the most common adverse effects, limiting treatment adherence in some patients [81]. Nevertheless, the clinical success of gefapixant provides proof of concept that selective modulation of ATP-mediated sensory signaling can effectively treat disorders characterized by neuronal hypersensitivity.

#### 7.3.2. Eliapixant

Eliapixant is a highly selective P2X3 antagonist developed to improve receptor specificity while minimizing taste-related adverse effects observed with earlier compounds [22]. Phase II clinical studies have demonstrated encouraging efficacy in chronic cough with a substantially lower incidence of dysgeusia compared with gefapixant [22]. The improved selectivity of eliapixant makes it an attractive candidate for chronic pain conditions requiring long-term treatment, including disorders associated with persistent visceral hypersensitivity [82]. Although newer P2X3 antagonists were developed to reduce taste-related adverse effects, dysgeusia has not been completely eliminated [83]. This may reflect residual inhibition of P2X2/3 receptors at therapeutic doses, a potential role of P2X3 receptors in taste signaling, and differences in receptor occupancy, pharmacokinetics, and individual susceptibility [84]. These observations suggest that receptor selectivity alone may not fully predict tolerability and warrant further investigation.

#### 7.3.3. Sivopixant

Sivopixant is another next-generation P2X3 antagonist designed to retain therapeutic efficacy while improving tolerability through greater receptor selectivity [23]. Clinical studies have reported reductions in cough frequency with relatively few taste disturbances, suggesting that selective inhibition of P2X3 receptors without significant P2X2/3 blockade may improve the overall safety profile [18]. Although its clinical evaluation has focused primarily on chronic cough, the underlying mechanism of sensory neuron inhibition supports potential application in chronic pelvic and visceral pain syndromes.

#### 7.3.4. Camlipixant

Camlipixant is a potent and highly selective P2X3 antagonist currently undergoing clinical evaluation for refractory chronic cough and other hypersensitivity disorders [24]. Early clinical studies have demonstrated meaningful reductions in cough frequency with improved tolerability compared with less selective antagonists [85]. The favorable balance between efficacy and adverse effects highlights the continued refinement of P2X3-targeted therapies and supports further investigation of this drug class for disorders characterized by peripheral sensory neuron hyperexcitability [86].

#### 7.3.5. Filapixant

Filapixant is a potent and highly selective P2X3 receptor antagonist that has been investigated for the treatment of refractory chronic cough [87]. In a phase 2 randomized crossover trial, filapixant significantly reduced cough frequency and severity while improving cough-related quality of life, supporting the role of P2X3 inhibition in attenuating sensory neuron hyperexcitability [88]. Although the drug was generally well tolerated, dose-dependent taste disturbances remained common, particularly at higher doses, suggesting that receptor selectivity alone may not fully eliminate this class-related adverse effect [88]. Nevertheless, the demonstrated clinical efficacy of filapixant further validates P2X3 receptor antagonism as a promising therapeutic strategy for disorders characterized by peripheral sensory hypersensitivity [89].

Collectively, the clinical development of P2X3 antagonists demonstrates that ATP-mediated sensory signaling is a viable therapeutic target [90]. Although these agents have primarily been developed for chronic cough, the shared mechanisms of peripheral sensitization, neuroimmune activation, and neuronal hyperexcitability observed in endometriosis and IBS suggest considerable potential for drug repurposing [91]. As summarized in Table 2, currently available P2X3 antagonists—including gefapixant, eliapixant, sivopixant, and camlipixant—have advanced through Phase II to IV clinical development and exhibit favorable safety profiles, with taste disturbance (dysgeusia) representing the principal adverse effect [22,23,24,81,87,90]. Future clinical trials should evaluate whether selective P2X3 inhibition can reduce chronic pelvic and visceral pain, identify patient populations most likely to benefit, and determine whether combination therapy targeting complementary nociceptive pathways provides greater clinical efficacy than P2X3 inhibition alone.

### 7.4. Drug Repurposing Opportunities in Endometriosis and IBS

The clinical development of P2X3 antagonists for refractory chronic cough provides a promising opportunity for drug repurposing in endometriosis and IBS, which share features of sensory neuron hyperexcitability, neuroimmune activation, and visceral hypersensitivity [90]. By selectively inhibiting ATP-mediated afferent pain signaling, P2X3 antagonists may offer a nonhormonal, mechanism-based approach for managing chronic pelvic and abdominal pain, particularly in patients with persistent symptoms after surgery, predominant visceral hypersensitivity, or overlapping endometriosis and IBS. Successful clinical translation will require careful patient selection, biomarker-guided stratification, and evaluation using pain-specific outcomes, while combination therapies targeting complementary pathways such as TRPV1, NGF, CGRP, hormonal signaling, or the microbiome may further improve therapeutic efficacy [65,66].

### 7.5. Current Challenges and Safety Considerations

Despite growing interest in P2X3 antagonists, several challenges remain before their integration into the management of endometriosis and IBS. Most evidence supporting ATP–P2X3 signaling is derived from preclinical studies, while direct clinical evidence of P2X3 upregulation in patients remains limited [37,41,90]. Chronic pelvic and visceral pain are also multifactorial, involving hormonal influences, inflammation, neuropathic mechanisms, myofascial dysfunction, and interacting pathways such as TRPV1, NGF, CGRP, ASICs, prostaglandins, and cytokines. Consequently, P2X3 inhibition alone may not provide complete symptom relief, particularly in patients with established central sensitization, highlighting the need for biomarker-guided patient selection and combination therapeutic strategies [36].

Additional challenges include optimizing dosing, treatment duration, predictors of response, and clinical endpoints, as well as establishing long-term safety, particularly for newer, more selective P2X3 antagonists developed to minimize taste disturbances associated with first-generation agents [86]. The effects of hormonal fluctuations, disease stage, and coexisting disorders of gut–brain interaction also require further investigation. Nevertheless, the clinical success of P2X3 antagonists in refractory chronic cough demonstrates that ATP-mediated sensory signaling is a clinically actionable pathway, providing a strong rationale for biomarker-driven clinical trials evaluating selective P2X3 inhibition as a precision therapy for endometriosis- and IBS-associated chronic pain [90].

## 8. Future Directions

### 8.1. Biomarker Discovery

The successful clinical translation of P2X3-targeted therapies will depend on the identification of reliable biomarkers that reflect purinergic pathway activation and predict therapeutic response [92]. Current evidence is insufficient to determine which patients with endometriosis or IBS exhibit clinically significant ATP–P2X3 dysregulation. Future studies should investigate circulating inflammatory mediators, extracellular ATP levels, purinergic receptor expression, transcriptomic signatures, and neuroimaging biomarkers as potential tools for patient stratification. The integration of molecular biomarkers with quantitative sensory testing and patient-reported outcomes may facilitate earlier diagnosis and improve treatment selection.

### 8.2. Precision Medicine Approaches

Endometriosis and IBS are biologically heterogeneous disorders, suggesting that not all patients will respond similarly to P2X3 inhibition. Precision medicine approaches integrating clinical phenotypes, molecular biomarkers, genomic profiling, and disease endotypes may identify patient populations most likely to benefit from targeted therapy [57]. Individuals with overlapping endometriosis and IBS, persistent visceral hypersensitivity, or evidence of enhanced neuroimmune activation may represent particularly suitable candidates for future clinical trials [27,28]. Combining P2X3 antagonists with hormonal therapies, neuromodulators, biologics, or microbiome-directed interventions may further improve therapeutic outcomes by targeting multiple mechanisms involved in chronic pain [93].

### 8.3. Knowledge Gaps and Research Priorities

Despite growing evidence implicating ATP–P2X3 signaling in chronic pain, several important knowledge gaps remain. Most available data originate from experimental models, whereas direct clinical evidence demonstrating P2X3 upregulation in patients with endometriosis, IBS, or coexisting disease is limited [77,93]. Large, prospective translational studies are needed to validate the role of purinergic signaling in disease pathogenesis and determine whether P2X3 receptor expression correlates with symptom severity, disease progression, or treatment response. Future randomized clinical trials should evaluate selective P2X3 antagonists in endometriosis and IBS using standardized pain measures, quality-of-life assessments, biomarkers of neuroimmune activation, and objective indices of visceral hypersensitivity. Addressing these gaps will determine whether ATP–P2X3 signaling represents not only a shared mechanistic pathway but also a clinically actionable target capable of improving personalized management of chronic pelvic and visceral pain.

## 9. Limitations of Current Evidence

Although accumulating evidence supports a role for ATP–P2X3 signaling in chronic pelvic and visceral pain, most mechanistic data are derived from animal models and in vitro studies, with relatively limited clinical evidence directly evaluating P2X3 receptor expression or activity in endometriosis and IBS. Furthermore, substantial heterogeneity in experimental models, disease induction methods, diagnostic criteria, pain assessment, and laboratory techniques, together with small sample sizes and predominantly cross-sectional study designs, limits direct comparisons and the translation of preclinical findings to clinical practice. Chronic pelvic and visceral pain also arise from multiple interacting mechanisms involving TRPV1, NGF, CGRP, ASICs, inflammatory cytokines, hormonal influences, and gut microbiota alterations, indicating that ATP–P2X3 signaling represents one component of a complex neuroimmune network rather than a single causal pathway [15,59,67].

Clinical evidence supporting the therapeutic use of P2X3 antagonists in endometriosis and IBS remains limited. Table 3 summarizes the current evidence available. Although these agents have demonstrated efficacy in refractory chronic cough, their effectiveness, optimal dosing, long-term safety, and appropriate patient selection for chronic pelvic and visceral pain have yet to be established. Nevertheless, the available evidence consistently identifies ATP–P2X3 signaling as a key contributor to neuroimmune pain mechanisms, supporting further translational studies and randomized clinical trials to determine whether selective P2X3 inhibition can be developed into a precision therapy for patients with endometriosis and IBS.

## 10. Conclusions

Endometriosis and IBS frequently coexist and share key pathophysiological features, including neuroinflammation, visceral hypersensitivity, peripheral and central sensitization, and neuroimmune activation. Current experimental evidence suggests that ATP-mediated activation of P2X3 receptors contributes to these processes by enhancing sensory neuron excitability and amplifying nociceptive signaling, supporting ATP–P2X3 signaling as a plausible shared mechanism underlying chronic pelvic and visceral pain.

Selective P2X3 antagonists have demonstrated the therapeutic potential of targeting purinergic signaling and may offer a promising nonhormonal, nonopioid approach for treating chronic pain in endometriosis and IBS. However, clinical translation remains limited by the lack of robust human data, highlighting the need for studies validating P2X3 receptor expression, identifying predictive biomarkers, and establishing long-term efficacy and safety. Collectively, ATP–P2X3 signaling provides a biologically plausible framework linking endometriosis and IBS and represents one of the most promising emerging targets for precision therapies aimed at chronic pelvic and visceral pain.

## Figures and Tables

**Table 1:** Summary of experimental studies supporting ATP–P2X3 receptor signaling as a shared neural sensitization pathway in endometriosis and IBS.

Study	Experimental Model/ Tissue	Study Type	Main Findings	Relevance to Shared P2X3 Neural Sensitization in IBS and Endometriosis	Key Limitations
Liñán-Rico, 2015 [69]	Human submucous plexus	Ex vivo human tissue	Identified ATP-gated P2X3 and other P2X receptors involved in excitatory purinergic signaling in enteric neural pathways.	Supports the involvement of ATP-mediated purinergic signaling in gastrointestinal nociception and visceral hypersensitivity in IBS.	Mechanistic ex vivo study; no clinical correlation or IBS-specific patient outcomes.
Ding, 2017 [70]	Human eutopic and ectopic endometrial tissues; cultured stromal cells	Human observational and in vitro	Demonstrated increased endogenous ATP concentration and elevated P2X3 receptor expression in endometriotic lesions and DRG tissues, positively correlated with hyperalgesia severity. Also identified ATP-activated P2X3 receptors in endometriosis-associated cells.	Suggests ATP-P2X3 signaling contributes to peripheral and central sensitization in endometriosis and may overlap mechanistically with IBS pain pathways.	Cross-sectional human tissue study; cannot establish causality.
Ding, 2020 [53]	Rat endometriotic lesions; DRG and spinal dorsal horn	In vivo rat model	Reported elevated P2X3 receptor expression in DRG tissues and ERK-mediated P2X3 upregulation in the spinal dorsal horn.	Supports DRG and spinal cord plasticity as contributors to chronic pelvic pain and cross-organ sensitization.	Animal model requiring validation in humans.
Trapero, 2019 [71]	Ectopic endometriotic lesions	Ex vivo human tissue	Found impaired ectonucleotidase activity causing extracellular ATP accumulation within ectopic lesions.	Suggests dysregulated ATP metabolism sustains ATP-mediated peripheral sensitization and chronic nociceptive signaling.	Observational tissue study; ATP dysregulation inferred from tissue analyses.
Y. Chen, 2015 [72]	Inflammatory pain model examining p38 signaling	In vivo/ex vivo animal study	Activation of p38 signaling reduced P2X3 receptor expression in neuronal somata, limiting receptor transport to peripheral and central nerve terminals and decreasing pain transmission.	Demonstrates intracellular signaling pathways regulate P2X3-mediated nociceptive sensitization.	General inflammatory pain model rather than IBS- or endometriosis-specific.
Jiang, 2017 [41]	Inflammatory rat model	In vivo rat model	ERβ agonists reduced P2X3 receptor expression in DRG neurons and reversed mechanical hyperalgesia. ERK antagonism also decreased hyperalgesia and ERK expression in DRG and spinal dorsal horn.	Highlights hormonal and ERK-mediated regulation of P2X3 receptors in chronic pain sensitization relevant to endometriosis and IBS overlap.	Findings derived from experimental inflammation models rather than human disease.
Weng, 2015 [73]	IBS-related visceral hypersensitivity rat models	In vivo rat model	Elevated ATP signaling was associated with activation and upregulation of P2X3 receptors during visceral hypersensitivity.	Provides direct evidence linking ATP-P2X3 signaling to IBS visceral pain mechanisms.	Experimental IBS model may not fully reproduce human IBS.
Z. Zhang, 2021 [74]	IBS chronic visceral hyperalgesia rat models	In vivo rat model	Observed increased P2X3 protein expression in the spinal cord, anterior cingulate cortex (ACC), and ventral posterolateral thalamus (VPL).	Suggests P2X3 receptors contribute to central sensitization, neuronal hyperexcitability, and maladaptive neuroplasticity in chronic pain disorders.	Small experimental groups; animal findings require clinical validation.
Sun, 2025 [75]	Chronic visceral pain induced in mice	In vivo mouse model	PSN-derived exosomal miR-1306-3p activated P2X3 receptors in spinal dorsal horn neurons, enhancing synaptic transmission and visceral pain.	Expands the role of P2X3 receptors beyond peripheral nociception to include neuroplasticity and neuron-glia communication involved in persistent visceral hyperalgesia.	Preclinical mechanistic study without human validation.
Dong, 2022 [76]	TNBS-induced rat model of chronic colitis and bladder overactivity	In vivo rat model	Increased P2X3 receptor expression in DRG neurons; inhibition of P2X3 receptors alleviated bladder overactivity and visceral hypersensitivity.	Supports the concept of cross-organ pelvic sensitization mediated by P2X3 signaling between gastrointestinal and pelvic organs.	TNBS-induced colitis models inflammatory bowel disease more closely than primary IBS.
Davenport, 2021 [22]	Rodent inflammatory pain and uterine inflammation models	Preclinical (in vitro, ex vivo, and in vivo)	Selective P2X3 receptor antagonist eliapixant rapidly reduced mechanical hyperalgesia and neurogenic plasma extravasation following uterine inflammation.	Demonstrates that P2X3 receptors contribute to both nociceptive signaling and neurogenic inflammation, supporting ATP-P2X3 signaling as a shared pathway underlying sustained visceral hypersensitivity in IBS and endometriosis.	Predominantly preclinical; clinical efficacy not assessed.
Parke, 2024 [77]	Phase 2b randomized, placebo-controlled clinical trial in women with endometriosis-associated pelvic pain	Phase 2b randomized clinical trial	Selective P2X3 receptor antagonist eliapixant in women with surgically confirmed endometriosis-associated pelvic pain. The trial was terminated early because of a drug-induced liver injury signal, limiting efficacy assessment.	Provides the first clinical evaluation of selective P2X3 receptor antagonism in endometriosis. Although efficacy was not demonstrated, the study supports the translational investigation of ATP–P2X3 signaling as a therapeutic target and highlights the need for safer, more selective P2X3 antagonists for chronic pelvic and visceral pain.	Early trial termination prevented a definitive assessment of efficacy.

**Table 2:** Clinical development of P2X3 receptor antagonists and their potential relevance to endometriosis and IBS.

Drug	Target	Clinical Phase	Primary Indication	Major Adverse Effect	Relevance to Endometriosis/ IBS
Camlipixant [24]	Selective P2X3	Phase II	Refractory cough	Taste disturbance	High
Eliapixant [22]	Selective P2X3	Phase II	Refractory cough	Taste disturbance	High
Filapixant [88]	Selective P2X3	Phase II	Refractory cough	Taste disturbance	High
Gefapixant [81]	P2X3/P2X2/3	Phase IV	Refractory cough	Taste disturbance	High
Sivopixant [23]	Selective P2X3	Phase II	Refractory cough	Taste disturbance	High

**Table 3:** Current evidence supporting ATP–P2X3 signaling as a shared mechanism linking endometriosis and irritable bowel syndrome.

Research Question	Current Evidence	Level of Evidence
Is extracellular ATP increased?	Experimental studies consistently demonstrate increased ATP release in inflamed endometriotic and intestinal tissues, promoting nociceptor activation and neuroimmune signaling.	Strong preclinical evidence
Is P2X3 receptor expression increased?	Animal studies consistently report P2X3 upregulation in dorsal root ganglia and peripheral sensory neurons, whereas direct evidence in human endometriosis and IBS remains limited.	Strong animal evidence; limited human evidence
Does ATP–P2X3 signaling contribute to chronic pain?	Experimental studies demonstrate that ATP-mediated P2X3 activation promotes peripheral sensitization, DRG plasticity, central sensitization, and visceral hypersensitivity.	Strong preclinical evidence
Can P2X3 antagonists reduce pain?	Pharmacological inhibition consistently attenuates nociceptor excitability, inflammatory pain, and visceral hypersensitivity in animal models of endometriosis and IBS.	Strong preclinical evidence
Has this pathway been evaluated clinically?	P2X3 antagonists have demonstrated clinical efficacy in refractory chronic cough, validating ATP-mediated sensory signaling as a therapeutic target. A Phase 2b trial of eliapixant in endometriosis represents the first clinical evaluation, although it was terminated early because of safety concerns.	Early clinical evidence
Is ATP–P2X3 signaling a promising therapeutic target?	The available evidence supports ATP–P2X3 signaling as a biologically plausible, mechanism-based target for chronic pelvic and visceral pain, although clinical validation remains limited.	Promising
What are the major knowledge gaps?	Direct human mechanistic studies, validated biomarkers of purinergic activation, optimal patient selection, long-term safety, and randomized clinical trials in endometriosis and IBS are still needed.	Priority for future research

ATP, adenosine triphosphate; DRG, dorsal root ganglion; IBS, irritable bowel syndrome; P2X3, purinergic receptor P2X3.

## Data Availability

No new data were created or analyzed in this study. Data sharing is not applicable to this article.

## Abbreviations

The following abbreviations are used in this manuscript:

IBS	Irritable bowel syndrome
ATP	Adenosine triphosphate
TRPV1	Transient receptor potential vanilloid 1
NGF	Nerve growth factor
CGRP	Calcitonin gene-related peptide
ASICs	Acid-sensing ion channels
DAMP	Danger-associated molecular pattern
DRG	Dorsal root ganglion
